# Microbiota and the Immune System—Actors in the Gastric Cancer Story

**DOI:** 10.3390/cancers14153832

**Published:** 2022-08-08

**Authors:** Marek Majewski, Paulina Mertowska, Sebastian Mertowski, Konrad Smolak, Ewelina Grywalska, Kamil Torres

**Affiliations:** 1 2nd Department of General, Gastrointestinal Surgery and Surgical Oncology of the Alimentary Tract, Medical University of Lublin, 20-081 Lublin, Poland; 2 Department of Experimental Immunology, Medical University of Lublin, 20-093 Lublin, Poland; 3 Chair and Department of Didactics and Medical Simulation, Medical University of Lublin, 20-093 Lublin, Poland

**Keywords:** stomach cancer, immune subtype of gastric cancer, *Helicobacter pylori*, immune checkpoint, microbiota

## Abstract

**Simple Summary:**

Stomach cancer is one of the most commonly diagnosed cancers in the world. Although the number of new cases is decreasing year by year, the death rate for this type of cancer is still high. The heterogeneous course and the lack of symptoms in the early stages of the disease mean that the diagnosis is made late, which translates into a worse prognosis for such patients. That is why it is so important to analyze potential risk factors that may increase the risk of developing gastric cancer and to search for new effective methods of treatment. These requirements are met by the analysis of the composition of the gastric microbiota and its relationship with the immune system, which is a key element in the human anti-cancer fight. This publication was created to systematize the current knowledge on the impact of dysbiosis of human microbiota on the development and progression of gastric cancer. Particular emphasis was placed on taking into account the role of the immune system in this process.

**Abstract:**

Gastric cancer remains one of the most commonly diagnosed cancers in the world, with a relatively high mortality rate. Due to the heterogeneous course of the disease, its diagnosis and treatment are limited and difficult, and it is associated with a reduced prognosis for patients. That is why it is so important to understand the mechanisms underlying the development and progression of this cancer, with particular emphasis on the role of risk factors. According to the literature data, risk factors include: changes in the composition of the stomach and intestinal microbiota (microbiological dysbiosis and the participation of *Helicobacter pylori*), improper diet, environmental and genetic factors, and disorders of the body’s immune homeostasis. Therefore, the aim of this review is to systematize the knowledge on the influence of human microbiota dysbiosis on the development and progression of gastric cancer, with particular emphasis on the role of the immune system in this process.

## 1. Introduction

**Figure 1:**
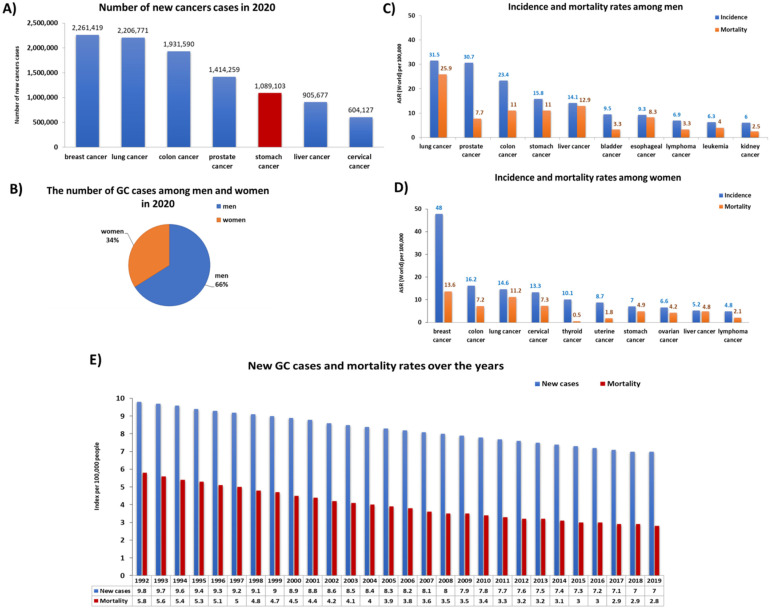
Statistics on the incidence and mortality of gastric cancer (GC) in the world. (**A**) Number of new cases cancer in 2020; (**B**) the number of GC cases among men and women in 2020; (**C**) incidence and mortality rates among men in 2020; (**D**) incidence and mortality rates among men in 2020; (**E**) new gastric cancer cases and mortality rates over the years (base on [4,5]) (ASR—Age-Standardized Rate).

Gastric cancer (GC) is one of the most commonly diagnosed cancer and the fifth leading cause of cancer-related death worldwide [1,2]. In 2018, it was estimated that this cancer was responsible for 780,000 deaths (8.8% of all cancer deaths) worldwide. According to data published by the World Health Organization (WHO), in 2020 the number of registered cases of GC in the world was 1,089,103 cases, which accounted for 5.6% of all cancer cases in the world. These data show that GC ranks fifth in the incidence of the leading cancer types (Figure 1A). A detailed analysis of these cases showed that men (66%) were significantly more frequent in GC than women (34%) (Figure 1B). The differences between the two genders concerned not only the incidence of GC, which was 2.25 times higher in men than in women, but also the number of deaths (Figure 1C,D). According to the literature data, despite the large number of registered new GC cases in the world, their number is decreasing year by year (Figure 1E). In the United States, incidence has decreased by 1.5% annually in the last decade [1,3].

Morbidity and mortality rates vary widely by geographic location, with well-defined high and low risk areas around the world [6]. More specifically, gastric cancer is common in East Asia, where nearly two-thirds of GC cases occur, Eastern Europe, and Latin America’s Pacific coast. In contrast, incidence rates are generally low in North America, South Asia, and Australia [7,8]. GC is most common in the elderly. About 60% of diagnosed people are over 65 [1,9]. The mean age of diagnosis is 68 years. The 5-year survival rate for people with gastric cancer is 32% [10]. This statistic reflects the fact that 62% of people with GC are diagnosed after the cancer has spread beyond its original site. If stomach cancer is found before it has spread, the 5-year survival rate is generally higher but depends on the stage of cancer found during surgery. According to the data presented by SEER (Surveillance, Epidemiology, and End Results Program) on patients diagnosed with gastric cancer in 2011–2017, the 5-year survival rates were as follows: localized tumors accounted for 70% of cases, regional tumors 32%, and distant neoplasms about 6% of cases [2]. Although GC incidence is declining in some developed societies thanks to appropriate interventions, it still remains a serious health threat, mainly in developing countries [11]. Due to the heterogeneity of the course and late diagnosis of GC, the prevention of this type of cancer is extremely difficult [12,13]. Identification and prevention of risk factors and underlying causes [14,15], such as changes in the composition of the gastric and intestinal microflora (microbiological dysbiosis and the participation of *Helicobacter pylori*), improper diet, environmental and genetic factors, and disorders of the body’s immune homeostasis. The immune system consists of two interconnected innate and adaptive arms, both of which have cellular and soluble effectors. The cells of the innate immune system react to foreign antigens, which are recognized by pathogen recognition receptors derived from different types of viruses, bacteria, or cancer cells. The innate immune system is evolutionarily conserved and acts as immune surveillance through cells such as macrophages, dendritic cells (DCs), neutrophils and NK cells, and soluble factors such as the complement system [16]. All these cells of the immune system are involved in maintaining immune homeostasis in the human body and fighting the developing inflammation or fighting pathogens. Chronic *H. pylori* infection leads to gastritis, and in some patients, peptic ulcer disease, and in about 1%—gastric cancer [17,18]. The likelihood of developing disease in those infected with these microorganisms is largely determined by the long-term inflammatory response that is associated with the virulence of the strains, genetic predisposition of the host, and environmental cofactors. The immune and inflammatory response to *H. pylori* infection is very important as gastritis may not only lead to further clinical consequences, an ineffective immune response, and increasing inflammation to persistent infection with these bacteria in the body but may also be the cause of the development of oncogenic processes within the stomach [19,20,21].

Due to the above facts, the aim of this publication was to systematize the knowledge on the impact of human microbiota dysbiosis on the development and progression of gastric cancer. Additionally, the review specifically analyzes the role of the immune system in the pathogenesis of this disease.

## 2. Materials and Methods

### Search Strategy, Study Selection, and Data Extraction

**Figure 2:**
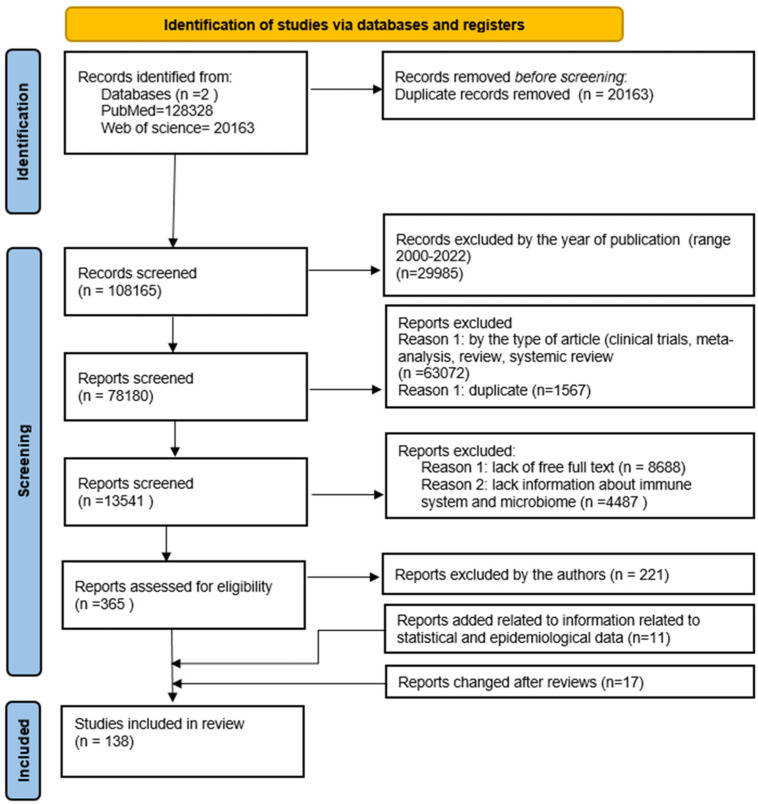
The flow diagram of the study’s inclusion and exclusion criteria.

The literature analysis was carried out on the PubMed database, where the search for available articles was performed based on the following keywords: “gastric cancer”, “gastric carcinoma”, “stomach cancer”. The time range of the searched articles was established for the years 2000 to 2022, and filters related to the type of articles (clinical trials, review, systematic review and metaanalysis) were used. Then, the remaining articles were filtered in terms of access to the full version and in terms of the occurrence of keywords such as “immune system and microbiome”, “microbiota”. The remaining articles were analyzed by the authors in terms of their inclusion in the publication. Duplicates were rejected at each stage of the analysis from among the articles found. Finally, 144 articles and 11 websites containing the necessary statistics were included. The scheme of the procedure is presented below (Figure 2).

## 3. Gastric Cancer—Risk Factors, Symptoms, and Classification

**Figure 3:**
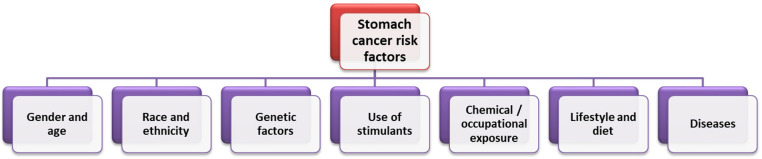
Factors influencing the incidence of GC (based on [24]).

Due to the multidimensional nature of GC and its heterogeneous classification, it is necessary for specialists to conduct research aimed not only at recognizing but also preventing or treating its risk factors, which may be an effective step in reducing the burden of this disease around the world [22]. Keep in mind that stomach cancers tend to develop slowly over years. Before true cancer develops, precancerous lesions often appear in the inner lining (mucosa) of the stomach. These early changes rarely cause symptoms, so they often go undetected until the late stages [23]. That is why it is so important to understand the factors influencing the development and progression of GC in modern society. As indicated in the literature, the pathogenesis of GC is influenced by many factors, such as sex, origin, genetic predisposition, lifestyle, and diet (Figure 3). Efforts to improve screening programs and the early detection and treatment of stomach cancer are important, but the priority is to take action to eliminate avoidable factors that play a significant role in the development of stomach cancer. Identifying the most important factors contributing to the development of stomach cancer and implementing preventive programs can prevent thousands of stomach cancer cases each year [24]. Therefore, it is extremely important to educate the public about GC risk factors and to introduce national and regional programs for monitoring and assessing cancer control plans.

### 3.1. Selected Risk Factors for the Development of Gastric Cancer

#### 3.1.1. Genetic Factors

The conducted research shows that some inborn gene mutations, such as the GSTM1-null phenotype (Glutathione S-Transferase Mu 1) or the CDH1 gene (Cadherin-1), increase the risk of developing GC. Loss of one copy of the CDH1 gene causes hereditary diffuse gastric cancer (HDGC), an autosomal dominant inheritance in which malignant cells pass under the lining of the stomach and subsequently metastasize [25,26,27,28]. Additionally, as indicated in the literature, genetic GC risk factors include the presence of polymorphisms in the genes encoding the interleukin IL-17 and IL-10, which are particularly common in Asian populations [29,30,31].

#### 3.1.2. Lifestyle and Diet

Salt-rich diet and the carcinogenic potential of known carcinogens such as N-methyl-*N*-nitro-*N*-nitrosoguanidine (MNNG) has been shown to be associated with an increased risk of stomach cancer [32,33]. This includes foods preserved by drying, smoking, salting, or pickling and foods that are high in salt. Eating salt-rich foods destroys the gastric mucosa, thus causing inflammation. There are links in the literature about cultures whose diets are high in salt and fermented foods, such as the Japanese, who have higher rates of stomach cancer. Moreover, from the available literature data it appears that Japanese immigrants in the United States who ingested and consumed Western food had a much lower GC incidence [34,35]. Preserved meat is rich in *N*-nitroso compounds, which can have a similar effect in the body to salt. Red meat is particularly rich in saturated fats and low in protective fats such as omega-3, which contributes to the development of inflammatory processes and thus increases the risk of stomach cancer [36,37]. Case-control studies showed that higher consumption of fruit and vegetables (rich in carotenoids, folates, phytochemicals, and vitamin C) was associated with a 37% lower risk of developing stomach cancer [38,39] Currently, diet modification is the best known form of GC prevention. A healthy diet rich in fruits, vegetables, whole grains and low in alcohol, pickles, and processed, smoked, or salted meat (especially red meat) not only reduces the risk of gastric inflammation but also reduces the risk of GC [40,41]. In addition, the use of an appropriate diet also helps to prevent hypertension and the development of obesity, thus reducing the risk of many chronic diseases [42].

#### 3.1.3. *H. pylori* Infection, Obesity, and other Diseases

The common bacterium *H. pylori* causes gastritis and ulcers. It is also considered to be one of the leading causes of stomach cancer. *H. pylori* tests are available and the infection can be treated with antibiotics [43,44]. More than half of the world’s population is infected with *H. pylori*, which can modulate the acidity of the stomach to alter the profile of the gastric microbiome, leading to *H. pylori*-related diseases. Moreover, there is increasing evidence that bacteria other than *H. pylori* and their metabolites also contribute to gastric carcinogenesis [45,46]. Therefore, elucidating the role of the gastric microbiome in the development and progression of GC can lead to improved prevention, diagnosis, and treatment. The incidence of *H. pylori* infection varies with age, ethnicity, and living conditions, with most cases occurring in childhood [47]. Only a small percentage of people develop pathological conditions associated with *H. pylori* infection, such as chronic gastritis, peptic ulcer, gastric adenocarcinoma, and gastric lymphoma (MALT). Chronic gastritis is an early manifestation of persistent inflammation caused by *H. pylori* infection. As the disease progresses, damage to the gastric epithelial cells can lead to the development of GC. *H. pylori* has been listed as a type I carcinogen by the World Health Organization (WHO) [48].

Apart from *H. pylori* infection, obesity is an important disease involved in the development of GC. A statistical meta-analysis from around the world showed that in people with excessive body mass index (BMI) (over 25 kg/m^2^), the probability of developing stomach cancer is 1.13 [49,50]. Along with increases in BMI, the strength of the disease relationship increased. Obesity was a particularly strong predisposing factor in men and non-Asians. Obesity can induce gastritis mediated by tumor necrosis factor-α (TNF-α), interleukin-6 (IL-6), and chemoattractive protein-1 (MCP-1). People on diets containing highly inflammatory foods, such as a diet high in meat and low in fruit and vegetables, have a higher risk of obesity [51,52,53,54].

The literature data also show that there is an increased risk of developing GC after gastric surgery [55]. The time lag between initial gastric surgery for mild disease and the development of gastric stump cancer is around 30 years or more, compared with 12 years if surgery was performed due to earlier detection of GC lesions [56]. Another medical condition that affects chances of developing GC is the occurrence of pernicious anemia or achlorhydria. The first medical condition occurs when the stomach cannot take in enough vitamin B12. This causes a severe drop in red blood cells [57]. On the other hand, the second disease occurs when there is no hydrochloric acid in the gastric juices, which helps to digest food [58].

### 3.2. Classification of Gastric Cancer

**Figure 4:**
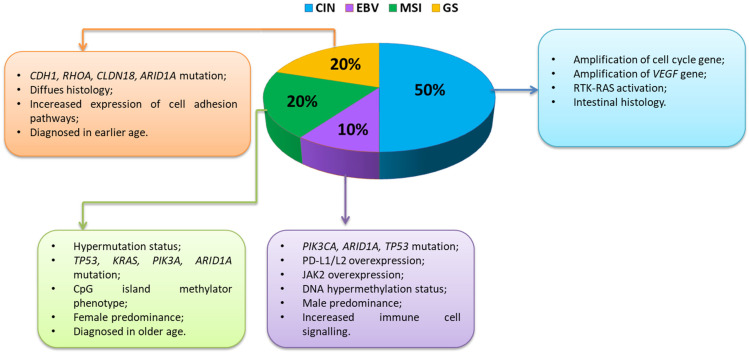
Characteristics of changes occurring within immunogenic GC subtypes (based on [69,70]) Abbreviations: CDH1—cadherin 1; RHOA—Ras homolog family member A; CLDN18—claudin 18; ARID1A—AT-rich interaction domain 1A; TP53—tumor protein P53; KRAS—Kirsten rat sarcoma virus; PIK3A—phosphatidylinositol-4,5-bisphosphate 3-kinase catalytic subunit alpha; PD-L1—programmed death-ligand 1; PD-L2—programmed death-ligand 2; JAK2—Janus kinase 2; VEGF—vascular endothelial growth factor; RTK-RAS—receptor tyrosine kinase-Ras.

Currently, in the literature, we can find three types of GC classification. The first is a basic clinical classification that distinguishes between early gastric cancer (EGC) and advanced gastric cancer (AGC). EGC is a lesion confined to the mucosa and submucosa, regardless of the presence of lymph node metastases. AGC is a lesion that crosses the submucosa, invading subsequent layers (muscle and serous membranes) and, in the next stage, adjacent organs. Due to the shallower EGC infiltration, it is associated with a better prognosis [59,60,61]. The second type is the histological classification developed and published by the WHO in 2018/2019, and it distinguishes five main types of gastric cancer: papillary, tubular, weakly coherent (including ring cancer), muscinic, and mixed as well as rare histological variants, such as neoplastic adenocarcinoma, squamous cell carcinoma, undifferentiated carcinoma, lymphoid stroma carcinoma, liver cancer, adenocarcinoma with enteroblast differentiation, gout adenocarcinoma, and microtubular adenocarcinoma [62,63]. A third type of GC classification under development is molecular classification based on genetic, epigenetic, and molecular signatures. The molecular characterization of GC has the advantage of offering a new tool for the development of targeted, more effective therapeutic strategies. Despite the obvious advantages of this classification, it is extremely difficult to compile. The cause of this condition is the heterogeneity of the disease itself as well as many biological mechanisms that may influence the pathogenesis of GC development [64,65]. The Cancer Genome Atlas (TCGA) conducted a groundbreaking study that used integrative genomics to molecularly phenotype four GC subtypes [66,67], which are to some extent related to the histological features of the disease: chromosomal instability (CIN), EBV-positive (EBV), microsatellite-unstable (MSI), and gnomically stable (GS). The TCGA study describes two specific subtypes, both of which are mainly composed of gut-type cancers with significant immunological linkages: the EBV subtype accounted for approximately 10% GC [67], which has a strong immune signature, and the MSI subtype (20%), which has a high mutational burden and also has a significant immune signature. For the other two subtypes: GS accounts for around 20% and the CIN subtype accounts for 50%, and this was different from the two previous immunogenic subtypes [68] (Figure 4).

The molecular, genetic, and immunological heterogeneity described by the TCGA emphasizes the need to stratify patients based on the likelihood of their response to various treatments, including immunotherapy. Nevertheless, many of the clinical trials described above have included patients with GC of all subtypes, which unfortunately may weaken the potential positive effects of these therapies. The EBV and MSI GC subtypes are associated with a strong immune response as well as over-expression of immune checkpoints, highlighting that these two GC subtypes are particularly attractive candidates for immune checkpoint blockade, and indeed research in these GC subtypes is ongoing. The EBV subtype described by TCGA is characterized by a high frequency of mutations within PIK3CA (phosphatidylinositol-4,5-bisphosphate 3-kinase catalytic subunit alpha), which suggests a possible therapeutic role of PI3K inhibitors (phosphoinositide 3-kinases). This subtype is also associated with the frequent occurrence of DNA hypermethylation and amplification in the *CD274* and *PDCD1LG2* (programmed cell death 1 ligand 2) genes, which encode the immunosuppressive proteins PD-L1 and PD-L2, emphasizing that this subtype is an ideal candidate for immunotherapy [69,71] (Figure 4).

The GS subtype consisted mainly of tumors classified as diffuse GC, with poorer survival compared to the Lauren gut type, and was associated with mutations in the CDH1 (cadherin-1) and RHOA (Ras homolog family member A) genes as well as with aneuploidy (Figure 4). The immunogenic GC subtypes EBV and MSI are likely more prominent to the immune system due to the expression of more neoantigens and other foreign epitopes that stimulate a strong immune response that can be enhanced with current therapies, while the less immunogenic GC, CIN, and GS subtypes are more hidden, and lower antigen presentation provides a stronger defense system against host immune attack [16,72]. To improve the efficacy of GC immunotherapy, new criteria based on different molecular and immunological subtypes are needed to predict potential response and prognosis [69].

### 3.3. Symptoms of GC

**Figure 5:**
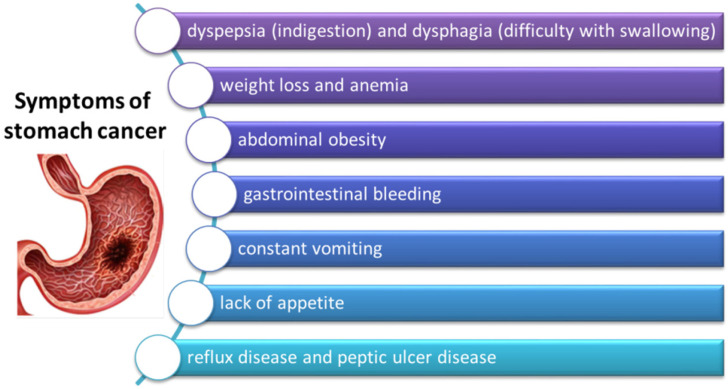
Symptoms of gastric cancer (based on [79]).

The early stage of the disease is often asymptomatic; therefore, the diagnosis is usually made in the advanced stage of the disease. Dyspeptic symptoms, as well as alarm symptoms that usually identify high-risk patients, occur not only in patients reporting to the general practitioner but also in the general population [73,74]. Gastroesophageal reflux disease, peptic ulcer disease, and functional dyspepsia are the most common causes of dyspeptic symptoms. Only in a few cases are the causes of dyspeptic symptoms malignant tumors of the stomach and esophagus [74]. Therefore, it is very important to carefully select patients at increased risk of gastro-esophageal cancer who should undergo endoscopy without delay. Alarm symptoms such as weight loss, dysphagia, signs and symptoms of upper gastrointestinal bleeding, anemia, and persistent vomiting (Figure 5) are probably more commonly associated with upper gastrointestinal malignancies, and most guidelines recommend immediate endoscopy in all patients with such symptoms [75,76]. However, the evidence for the presence of alarm symptoms as selection criteria for endoscopy is inconsistent as, on the one hand, these symptoms are not sensitive enough to detect malignant neoplasms, and, on the other hand, their overall prevalence in the dyspeptic population is high. While the incidence of cancer is high in the dyspeptic population, the incidence of cancer of the digestive tract is very low. The studies conducted so far describing the high frequency of alarm symptoms in malignant neoplasms of the gastrointestinal tract are mainly retrospective and, according to these studies, up to 90% of patients with malignant neoplasms of the stomach and esophagus experience alarm symptoms during endoscopy [77,78]. Less significant results were obtained in large prospective cohort studies. Each year, approximately 3–4% of the population in industrialized countries presents to their GP with upper gastrointestinal symptoms, of which over 10% have alarm symptoms [73,79]. The symptoms of GC correlate well with the stage of the cancer at diagnosis and are probably also prognostic. Several studies have assessed the prognostic value of specific alarm symptoms in gastric cancer, showing that they may be independently related to the survival of gastric cancer patients, and that an increased number of alarm symptoms and specific symptoms are closely correlated with the risk of death. Studies assessing the impact of these symptoms on the survival of patients with gastric cancer have shown that the presence of at least one of them may reduce 5-year survival by an average of 26% [80]. However, obtaining complete certainty about the prognostic value of alarm symptoms remains a dispute among researchers due to different criteria for defining symptoms, retrospective data collection, geographic differences, and the age of patients enrolled in the study [80].

## 4. The Role of the Microbiome in the Development and Progression of GC

Microbiota, understood as a collection of microorganisms (all bacteria, archaea, eukaryotes and viruses) present in a specific environment, significantly contribute to trophic functions, metabolism, barrier function, immunological stimulation, and the signaling of virtually all organs of the human body [81]. Each organ has its own and often unique composition of microorganisms that live there and contribute significantly to the health of the body. At the same time, the influence of the environment, genetic predisposition, or our lifestyle and diet can significantly disturb the microbiota, contributing to the development of many diseases as well as participate in the process of carcinogenesis [82,83]. In this part of the article, we focus on the role of symbiosis and dysbiosis in the stomach microbiome and how changes in the composition of microorganisms affect the development or progression of this condition.

### 4.1. Stomach as a Living Environment for Microorganisms

The stomach is an extremely specific ecological niche inhabited by a few microorganisms that are highly adaptable to changing environmental conditions. The literature data estimate that the number of bacteria in the stomach is less than 10 CFU/g content compared to the duodenum, at 10^1^–10^9^ CFU/g, or the large intestine, with approximately 10^10^–10^12^ CFU/g [84]. First of all, the presence of gastric acid means that only a few microorganisms are able to survive in this unfavorable environment. We can distinguish here *H. pylori, Lactobacillus,* and *Streptococcus* and yeast *Candida albicans* fungi. In a situation where there is the development of neoplastic changes in the stomach or inflammation, we observe a clear increase in pH, which means a modification of the microenvironment and the possibility of colonization of the organ by a greater number of microorganisms [85]. However, scientists still do not agree whether the gastric microbiota associated with the presence of lower acidity is significantly associated with the pathogenesis of GC.

### 4.2. Symbiosis and Dysbiosis of the Stomach Microbiota

Currently, in the literature, the concept of microbiota symbiosis (eubiosis) and dysbiosis is dominant in the context of describing the state of intestinal microorganisms. The term dysbiosis is a broad term that defines the imbalance of the intestinal microflora in which we observe the loss of the beneficial contribution or signal of microorganisms with the simultaneous expansion of pathogenic microorganisms. Dysbiosis is believed to cause pro-inflammatory effects and immune dysregulation associated with various disease states, including carcinogenesis [86]. However, this term may also refer to other organs in the human body in which we observe similar relationships, including the stomach. The gastric mucosa and its composition of the microbiome are not fully understood by scientists. This is mainly due to the lack of easy access as well as significant differences within the population (resulting from ethnic origin, age, and different food patterns), which significantly affect the results of the analyses. Therefore, most of the studies available in the literature compare the microbiological differentiation of the stomach in the context of samples collected from patients diagnosed with GC with healthy patients [50,87].

**Figure 6:**
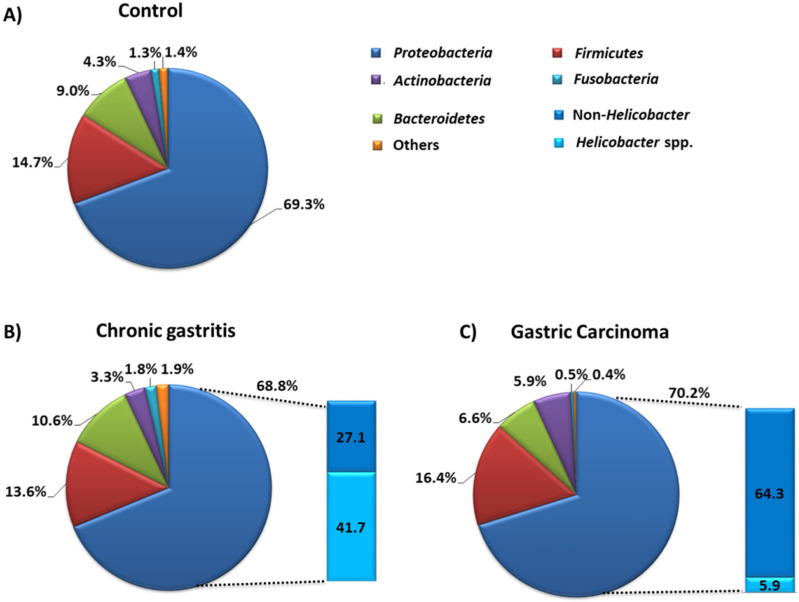
Composition of stomach microbiota in the state of dysbiosis and under the control conditions. (**A**) Composition of stomach microbiota in control group of patients. (**B**) Composition of stomach microbiota in chronic gastritis. (**C**) Composition of stomach microbiota in gastric carcinoma (based on [88]).

Overall, in the gastric microflora, scientists found that five types of microorganisms predominate: *Proteobacteria*, *Firmicutes*, *Bacteroidetes*, *Actinobacteria,* and *Fusobacteria* (Figure 6A). The analysis of the composition of the gastric microbiota in pathological conditions, especially in relation to the protection of gastritis and GC, showed that there was a significant difference in the number of the dominant types of microorganisms. The research conducted by the team of Ferreira et al., in 2017 [88], showed that in the case of chronic gastritis we can observe a decrease in the number of *Actinobacteria* (by 1%), *Firmicutes* (by 1.1%), and *Proteobacteria* (by 0.5%), with an increase in *Bacteroidetes* (by 1.6%) and *Fusobacterium* (by 0.5%), compared to controls (Figure 6A,B). Changes in the composition of the microbiota were also visible in samples from patients diagnosed with GC. There was a decrease in the number of *Bacteroidetes* (by 2.4%) and *Fusobacterium* (by 0.8%), as well as an increase in *Proteobacteria* (by 0.9%), *Firmicutes* (by 1.7%), and *Actinobacteria* (by 1.6%) compared to the control (Figure 6A,C). Moreover, significant differences in the composition of the gastric microbiota also concerned the disease states themselves. In the course of GC, we observe an increased number of *Proteobacteria* (by 1.4%), *Actinobacteria* (by 2.6%), *Firmicutes* (by 2.8%) and a decrease in the number of *Bacteroidetes* (by 4%) and *Fusobacterium* (by 1.3%) (Figure 6B,C). At the same time, an extremely important difference concerns the differentiation of the type of *Proteobacteria* itself, which is divided into bacteria belonging to *Helicobacter* spp. and bacteria not belonging to *Helicobacter* (non-Helicobacter), such as species *Phyllobacterium* and *Archomobacter* or families *Xanthomonadaceae* or *Enterobacteriaceae*. Ferreira’s team showed that the amount of *Helicobacter* bacteria is seven times lower in GC than chronic gastritis, which is also accompanied by an over twofold increase in the number of bacteria not classified as *Helicobacter* spp. (Figure 6B,C).

Statistical analysis also showed that the relative abundance of Helicobacter bacteria in the course of chronic gastritis was inversely correlated with the abundance of other *Proteobacteria*, *Firmicutes*, and *Bacteroidetes* types. In the case of GC, the abundance of Helicobacter was correlated with the abundance of *Bacteroidetes* and *Fusobacteria*. The results presented by the Ferreira team show that in disease states the abundance and diversity of the gastric microbiota undergoes significant changes, and the *Helicobacter* itself in the course of GC is not very numerous [88].

#### The Role of Gastric Microbiota Metabolites in the Pathogenesis of GC

Nitrogen compounds (NOC) involved in the process of carcinogenesis in the human body may come from two main sources. The first is a diet rich in processed meat, smoked fish, or some vegetables or fruits (exogenous source), while the second is oral microorganisms, which are able to reduce nitrates from food to nitrites, which are then converted in the stomach into NIGHT (endogenous source) [50]. Such microorganisms include bacteria of the following genera: *Clostridium, Haeomphilus, Staphylococcus, Veillonella, Nitrospirae,* or also *Lactobacillus* [89]. A study by the Jo team in 2016 shows that the presence of bacteria capable of reducing nitrates was higher in GC patients than in the control group [90]. In addition, these observations are confirmed by the research conducted by Ferreira in 2018. in which researchers found that nitrate and nitrite reductase activity was significantly higher in patients diagnosed with GC than in patients with chronic gastritis [91]. These studies suggest that changes in the stomach environment during the process of carcinogenesis not only change the pH values, allowing the increase of the diversity of the microenvironment but also facilitate the colonization of NOC-producing bacteria, which additionally contribute to the progression of cancer. An interesting phenomenon is the increase in the number of *Lactobacillus* bacteria, which are responsible for the synthesis of lactic acid, observed among GC patients. In the literature, we can find numerous works on the protective effect of this compound on the human body. However, lactic acid bacteria have a number of other mechanisms that can significantly promote carcinogenesis, including through increased production of ROC (reactive oxygen center) or NOC, which affect DNA damage and induce the mutagenesis process. In addition, NOC compounds produced by these microorganisms stimulate the expression of protooncogens and induce angiogenesis and inhibit the process of cell apoptosis [92,93,94]. The level of lactate also seems to be important in the pathogenesis of GC. Scientific research shows that lactate is treated by cancer cells as a source of energy and is involved not only in the processes of tumor development (angiogesis or escape from the immune system supervision) but also in the metastasis process (increased migration of tumor cells) [95,96]. Another metabolite that may affect the development of gastric cancer is short-chain fatty acids (SCFA), which are produced by the intestinal microbiota as a result of the fermentation of dietary fiber, acetate, propionate, and butyrate. Responsible for the production, among others such bacteria as *Bacteroides* spp., *Bifidobacterium* spp., *Ruminococcus* spp. (acetate synthesis), *Bacteroides* spp., *Coprococcus catus*, *Salmonella* spp. (propionate synthesis), and *Bifidobacterium, Propionibacterium, Lactobacillus* (butyrate synthesis) [97]. The above compounds support the intestinal barrier in mucus production and the regulation of tight-junction proteins [97]. SCFAs, such as acetate, propionate, and butyrate, are recognized as important factors in the mechanism of lipid metabolism through the interaction between glucagon-like peptide-1 (GLP-1) and GPR41 and GPR43 receptors on the surface of G protein-coupled cells. [97,98]. In colon cancer studies, these have an inhibitory effect on cell proliferation and the induction of apoptosis in neoplastic cells and possibly also in gastric cancer [99]. In addition, SCFAs also stimulate the differentiation of T cells that secrete IL-17, IFN-γ, and IL-10 and therefore may indirectly participate in the regulation of pro-inflammatory and anti-inflammatory responses [100]. Moreover, an increased incidence of inflammatory disorders has been observed in patients with breast cancer and gastric cancer who had a diet low in SCFA. It is also believed that the presence of SCFA in the host’s digestive system may positively affect the treatment of cancer and slow down the process of carcinogenesis [101].

## 5. The Role of Selected Microorganisms and Immunity in the Development and Progression of Gastric Cancer

**Figure 7:**
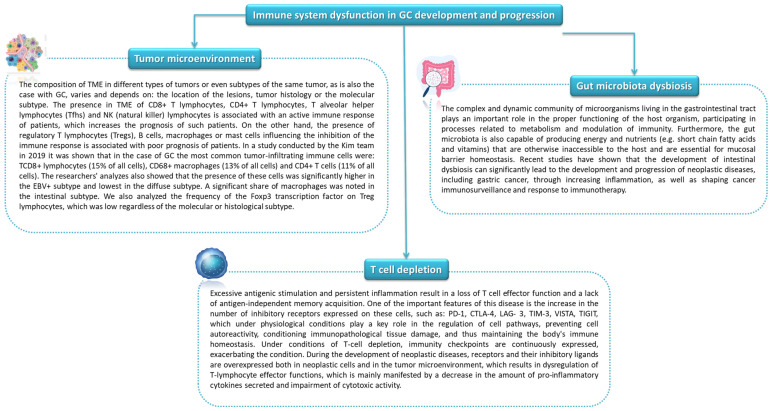
Immune system dysfunction in GC development and progression (based on [105,106,107,108,109]).

Inflammation has a significant impact on the host’s defense against pathogens as well as in the processes of repair, regeneration, and tissue remodeling. As it extends in the body, pathological conditions, including carcinogenesis, develop. Inflammation promotes cancer progression primarily by blocking antitumor immunity as well as shaping the tumor microenvironment towards a more favorable tumor cell and by exerting direct signals and tumor promoting functions [102,103,104]. Immune system disorders in the course of GC include changes in the microenvironment of the tumor itself as well as immune depletion of T lymphocytes and the involvement of immune checkpoints [20] (Figure 7).

### 5.1. The Role of Selected Microorganisms and Inflamantion

**Figure 8:**
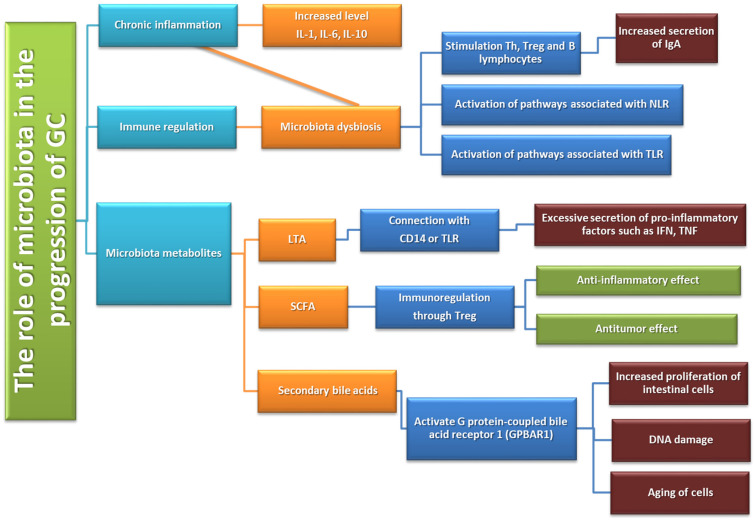
Immune system dysfunction in GC development and progression based on [111].

Inflammation affects the activation, recruitment, and function of many cells of the immune system related to both innate and acquired immunity. One of the pathways causing inflammation may be the activation of an inflammatory response through TLRs stimulated by Gram-negative bacteria. As a result of activation by bacterial lipopolysaccharide (LPS), the TLR receptor induces the NF-κB factor which, due to the involvement of genes responsible for the inflammatory response, enables the triggering of an innate and adaptive response [110]. Despite the benefits of activating this type of mechanism, its long-term action may lead to the development of pathological conditions, including carcinogenesis. Chronic inflammation promotes tumor progression mainly by blocking anti-tumor immunity as well as shaping the tumor microenvironment towards a more favorable tumor cell and by exerting direct signals and tumor promoting functions [102,103,104] (Figure 8).

One of the microorganisms that may contribute to the development of inflammation is *H. pylori*, which has developed a number of mechanisms allowing the colonization of the human stomach. *H. pylori* is one of the best known microorganisms that are active stimulators of the immune response [110]. Thanks to the production of urease, catalase, and oxidase, it is able to neutralize the acidic environment of the gastric juice and adhere to the gastric epithelium, which is crucial in avoiding the host’s immune response [112]. *Helicobacter* strains are equipped with various virulence factors that directly or indirectly influence the development of carcinogenesis [113,114]. First of all, they are the two proteins VacA and CagA. The former has the characteristic ability to induce vaculosis in epithelial cells and is toxic to various types of host cells. It is also involved in the process of weakening gastric epithelial cells by increasing the permeability of the mitochondrial membrane, disrupting transport or inducing apoptosis. It also has the ability to modulate the immune response by inhibiting the proliferation of immune cells and stimulate the production of pro-inflammatory cytokines (TNF-α or IL-6) by mast cells, which contributes to the development of inflammation in the stomach [115,116,117]. The second protein, CagA, interacts with gastric epithelial cells, inducing inflammation, which is strongly associated with the development of the carcinogenesis process. In addition, this protein is involved in the induction of proliferation and inhibition of apoptosis as well as in disorders of gastric epithelial continuity by disrupting intercellular connections or causing loss of cell polarity [118,119]. Chronic inflammation causes the loss of parietal cells that produce gastric acid, leading to a decrease in pH, which is significantly associated with changes in the amount and diversity of the microbiota. The changes in the gastric microbial profile may significantly increase the risk of GC incidence [120]. Moreover, both the presence of *H. pylori* and gastric inflammation lead to the production of significant amounts of reactive oxygen and nitrogen species, which are responsible for DNA damage (due to point mutations or double-stranded DNA breakage) as well as dysregulation of signaling pathways and induction of apoptosis or gastric epithelial cell autophages [121,122]. The role of the immune response in response to *H. pylori*-induced inflammation is the subject of many studies available in the literature. There is an increase in the expression of many inflammatory mediators (IL-6, IL-8, IL-1β, TNF-α), which is accompanied by the recruitment of a number of cells of the immune system to the gastric mucosa, e.g., T and B lymphocytes, as well as neutrophils, macrophages and dendritic cells. Additionally, an increased expression of PD-L1 in gastric epithelial cells was observed in the course of *H. pylori* infection, which significantly influences the dysregulation of the immune response [112,123].

Another example of a microorganism involved in the pathogenesis of GC is the genus *Prevotella*. These anaerobic gram-negative pleomorphic rods have been detected as one of the dominant species in GC patient samples [124,125,126]. More specifically, there are two representatives of this type: *Prevotella acnes* and *Prevotella copri*. The first has been associated with lymphocytic gastritis, which is one type of chronic gastritis characterized by a dense infiltration of the surface and epithelium by T cells and accompanying chronic infiltration of the lamina propria [127]. The mechanism of action of this bacterial species is based on the activation of NKG2D (produced by NK cells, γδ T cells, and CD8+ αβ T cells in humans) and pro-inflammatory IL-15. The research presented by the Montalban-Arques team in 2016 shows that both NKG2D and IL-15 are actively secreted in the gastric mucosa of LyG patients, and the gastric epithelial cells themselves respond to stimuli from these microorganisms (including live *P. acnes*) [128]. Additionally, it has been shown that the NKG2D and IL-15 system are strongly associated with the development of carcinogenesis [129]. While the participation of *P. acnes* in the GC pathogen has been described by scientists, the role of the second species of *P. copri* is not fully understood. From research carried out by Liu in 2019, in 276 patients from China, it was shown that the presence of *P. coprii* decreased with the increase of *P. acnes* in tissues with a degenerate GC [130]. As indicated in the literature, another example of bacteria involved in the GC pathogen may be *Fusobacterium nucleatum*. It is a Gram-negative bacterium, commonly found in the human mouth, and is associated with the development of periodontitis but also plays an important role in the carcinogenesis process [131,132]. Boehm’s team in 2020 showed that the presence of *F. nucleatum* did not significantly affect the occurrence of chronic gastritis or precancerous conditions. However, its presence was associated with significantly worse overall survival in patients with diffuse Lauren GC (but not with GC). In their studies, they did not find a significant relationship between the presence of *F. nucleatum* and gender, the presence of *H. pylori*, or the stage or the location of neoplastic lesions. However, Boehm’s team showed that this bacterium was positively correlated with the age of patients and a tendency to lower DNA methylation [132]. A study by the Hsieh team in 2021 shows that about a third of GC patients were positive for *F. nucleatum*, and their statistical analysis showed that the risk of colonization is significantly increased in patients with advanced cancer stages. The researchers suggested that the presence of *F. nucleatum* leads to deregulation of actin dynamics and possibly contributes to altering the mobility of cancer cells. They also performed an analysis of patients’ survival versus the presence of *F. nucleatum*, which showed that colonization by this bacterium was associated with poorer survival in patients as well as the presence of a positive result for *H. pylori* [133]. Both research teams indicate the need for further research to determine the role of *F. nucleatum* in the pathogenesis of GC and to analyze the relationship of this microorganism with other gastric microbiota bacteria as well as the potential benefits of targeted therapy. Other microorganisms may also be involved in the pathogenesis of GC, but their role is not fully understood. Such microorganisms include *Stenotrophomonas*, which is a low pathogenic non-sporulating bacterium that causes opportunistic infections [134], as well as *Selenomonas* [135]. Both of these populations correlated positively with the occurrence of BDCA2+ pDCs and Foxp3+ Tregs in GC samples [135].

### 5.2. Influence of the Gut Microbiota on Anti-Tumor Immunity

Microbiota can also influence the anti-cancer response. Two of the microorganisms that have such an effect are *Bifidobacterium* and *B. fragilis*, which, when administered to patients, increased the effectiveness of treatment with the use of PD-L1 and CTLA-4 blockades. In the case of *Bifidobacterium*, it was noted that the activation of dendritic cells and the CD8 + T-cell response directed at cancer cells increased with the participation of these bacteria. In addition, a few studies indicate that *Bifidobacterium* is able to influence the intestinal microflora and its change so that it is dependent on Treg lymphocytes, which consequently translates into an improvement in intestinal Treg suppressive functions, thus reducing inflammation in the colon during treatment with blockade of CTLA-4 and PD-L1 [136]. At the same time, *B. fragilis* bacteria can activate Th1 lymphocytes [137]. Other microorganisms, such as *Prevotella* CAG: 485 and *Akkermansia*, are able to influence the effectiveness of immunotherapy with PD-1 inhibitors by modulating glycerophospholipid metabolism, which, as researchers suggest, may affect the level of IL-2 or IFN-γ expression. Additionally, it is indicated that microorganisms that produce SCFA as products of their metabolic transformations have better anti-PD-1/PD-L1 responses [138]. In the case of bacteria such as *Enterococcus hirae* and *Lactobacillus johnsonii*, it is indicated that their presence is important for the anti-tumor effects of cyclophosphamide (CTX). The presence of these microorganisms influenced the promotion of the Th1 spleen memory and the activation of the Th17 response. Moreover, in the case of chemotherapy, the microbiome may affect innate immunity by reducing the production of pro-inflammatory cytokines as well as by reducing the number of cells presenting the antigen [136]. At the same time, the antitumor role of microbiota still requires further research, a deeper understanding and an understanding of the relationship between the systemic influence of microbiota on the immune system and the role of microflora in the treatment of cancer, in particular cancer of the digestive system [137].

## 6. Conclusions

Gastric cancer remains one of the most commonly diagnosed cancers in the world, with a relatively high mortality rate. Due to the heterologous course of the disease, its diagnosis and treatment are limited and difficult, which is associated with a reduced prognosis of patients. That is why it is so important to understand the mechanisms underlying the development and progression of this cancer, with particular emphasis on the role of epidemiological, environmental, and genetic factors and the role of the immune system. More and more studies and literature reports indicate the important role of changes in gastric microbiological differentiation in the course of GC, which may be not only one of the determinants of disease development but also a potential therapeutic target. However, due to the limited access to gastric mucosa, as well as many factors and predispositions of the patients themselves, these tests are extremely complicated and their results are often heterogeneous, which significantly limits their therapeutic potential. That is why it is so important to develop new, standardized methods of examining the composition of the gastric microbiome, which will allow for the comparison of the obtained results between patient populations and molecular, histopathological, or immunological GC subtypes. Due to the accompanying inflammation of tumors, it is also important to take into account the role of the immune system in the pathogenesis of GC. Thanks to the development of molecular research, researchers are providing and possessing an increasing amount of significant information on the composition of immune cells in TNM and the expression of individual receptors on their surface. This allows for the analysis of the signal transduction pathway between individual cancer cells and healthy cells, especially in the context of their disorders and regulation mechanisms. The information provided presents the state of the immune fault in the fight against the disease and thus allows not only the estimation of patients’ prognosis but also the selection of appropriate therapeutic methods, including pharmacological methods of blocking immune checkpoints. However, to fully understand the mechanisms underlying the development and progression of GC, many interdisciplinary studies are still needed, which will allow for a comprehensive analysis of not only the composition of the microbiota and its effects on the human body but also insight into the functioning of the immune system of patients.

## Data Availability

Not applicable.

## References

[1] Stomach (Gastric) Cancer Key Statistics. https://www.cancer.org/cancer/stomach-cancer/about/key-statistics.html.

[2] Stomach Cancer-Statistics. https://www.cancer.net/cancer-types/stomach-cancer/statistics.

[3] Rawla P., Barsouk A. (2019). Epidemiology of Gastric Cancer: Global Trends, Risk Factors and Prevention. Prz. Gastroenterol..

[4] Cancer Today. http://gco.iarc.fr/today/home.

[5] Cancer of the Stomach-Cancer Stat Facts. https://seer.cancer.gov/statfacts/html/stomach.html.

[6] Shah S.C. (2021). Gastric Cancer: A Neglected Threat to Racial and Ethnic Minorities in the USA. Lancet Gastroenterol. Hepatol..

[7] Pereira C., Park J.-H., Campelos S., Gullo I., Lemos C., Solorzano L., Martins D., Gonçalves G., Leitão D., Lee H.-J. (2022). Comparison of East-Asia and West-Europe Cohorts Explains Disparities in Survival Outcomes and Highlights Predictive Biomarkers of Early Gastric Cancer Aggressiveness. Int. J. Cancer.

[8] Katoh H., Ishikawa S. (2021). Lifestyles, Genetics, and Future Perspectives on Gastric Cancer in East Asian Populations. J. Hum. Genet..

[9] Stomach Cancer Incidence Statistics. https://www.cancerresearchuk.org/health-professional/cancer-statistics/statistics-by-cancer-type/stomach-cancer/incidence.

[10] Stomach (Gastric) Cancer Survival Rates. https://www.cancer.org/cancer/stomach-cancer/detection-diagnosis-staging/survival-rates.html.

[11] Khatoon J., Rai R.P., Prasad K.N. (2016). Role of *Helicobacter pylori* in gastric cancer: Updates. World J. Gastrointest. Oncol..

[12] Carcas L.P. (2014). Gastric cancer review. J. Carcinog..

[13] Lee Y.Y., Derakhshan M.H. (2013). Environmental and Lifestyle Risk Factors of Gastric Cancer. Arch. Iran. Med..

[14] Yang L., Ying X., Liu S., Lyu G., Xu Z., Zhang X., Li H., Li Q., Wang N., Ji J. (2020). Gastric Cancer: Epidemiology, Risk Factors and Prevention Strategies. Chin. J. Cancer Res..

[15] Sitarz R., Skierucha M., Mielko J., Offerhaus G.J.A., Maciejewski R., Polkowski W.P. (2018). Gastric Cancer: Epidemiology, Prevention, Classification, and Treatment. Cancer Manag. Res..

[16] Wang M., Busuttil R.A., Pattison S., Neeson P.J., Boussioutas A. (2016). Immunological Battlefield in Gastric Cancer and Role of Immunotherapies. World J. Gastroenterol..

[17] Kumar S., Patel G.K., Ghoshal U.C. (2021). *Helicobacter Pylori*-Induced Inflammation: Possible Factors Modulating the Risk of Gastric Cancer. Pathogens.

[18] Zhang R.-G., Duan G.-C., Fan Q.-T., Chen S.-Y. (2016). Role of *Helicobacter Pylori* Infection in Pathogenesis of Gastric Carcinoma. World J. Gastrointest. Pathophysiol..

[19] Kwak Y., Seo A.N., Lee H.E., Lee H.S. (2020). Tumor Immune Response and Immunotherapy in Gastric Cancer. J. Pathol. Transl. Med..

[20] Chen Y., Sun Z., Chen W., Liu C., Chai R., Ding J., Liu W., Feng X., Zhou J., Shen X. (2021). The Immune Subtypes and Landscape of Gastric Cancer and to Predict Based on the Whole-Slide Images Using Deep Learning. Front. Immunol..

[21] Becerril-Rico J., Alvarado-Ortiz E., Toledo-Guzmán M.E., Pelayo R., Ortiz-Sánchez E. (2021). The Cross Talk between Gastric Cancer Stem Cells and the Immune Microenvironment: A Tumor-Promoting Factor. Stem. Cell Res. Ther..

[22] Hu B., El Hajj N., Sittler S., Lammert N., Barnes R., Meloni-Ehrig A. (2012). Gastric Cancer: Classification, Histology and Application of Molecular Pathology. J. Gastrointest. Oncol..

[23] Gullo I., Grillo F., Mastracci L., Vanoli A., Carneiro F., Saragoni L., Limarzi F., Ferro J., Parente P., Fassan M. (2020). Precancerous Lesions of the Stomach, Gastric Cancer and Hereditary Gastric Cancer Syndromes. Pathologica.

[24] Poorolajal J., Moradi L., Mohammadi Y., Cheraghi Z., Gohari-Ensaf F. (2020). Risk Factors for Stomach Cancer: A Systematic Review and Meta-Analysis. Epidemiol. Health.

[25] Zhao Y., Deng X., Song G., Qin S., Liu Z. (2013). The GSTM1 Null Genotype Increased Risk of Gastric Cancer: A Meta-Analysis Based on 46 Studies. PLoS ONE.

[26] Zhang X.-L., Cui Y.-H. (2015). GSTM1 Null Genotype and Gastric Cancer Risk in the Chinese Population: An Updated Meta-Analysis and Review. OncoTargets Ther..

[27] Hereditary Diffuse Gastric Cancer (HDGC). https://www.hopkinsmedicine.org/health/conditions-and-diseases/hereditary-diffuse-gastric-cancer-hdgc.

[28] Luo W., Fedda F., Lynch P., Tan D. (2018). CDH1 Gene and Hereditary Diffuse Gastric Cancer Syndrome: Molecular and Histological Alterations and Implications for Diagnosis and Treatment. Front. Pharmacol..

[29] Long Z.-W., Yu H.-M., Wang Y.-N., Liu D., Chen Y.-Z., Zhao Y.-X., Bai L. (2015). Association of IL-17 Polymorphisms with Gastric Cancer Risk in Asian Populations. World J. Gastroenterol. WJG.

[30] Chen L., Shi Y., Zhu X., Guo W., Zhang M., Che Y., Tang L., Yang X., You Q., Liu Z. (2019). IL10 Secreted by Cancer associated Macrophages Regulates Proliferation and Invasion in Gastric Cancer Cells via CMet/STAT3 Signaling. Oncol. Rep..

[31] Tang J., Pan R., Xu L., Ma Q., Ying X., Zhao J., Zhao H., Miao L., Xu Y., Duan S. (2021). IL10 Hypomethylation Is Associated with the Risk of Gastric Cancer. Oncol. Lett..

[32] Alizadeh A.M., Afrouzan H., Dinparast-Djadid N., Sawaya A.C.H.F., Azizian S., Hemmati H.R., Mohagheghi M.A., Erfani S. (2015). Chemoprotection of MNNG-Initiated Gastric Cancer in Rats Using Iranian Propolis. Arch. Iran. Med..

[33] Kim T.-J., Kim M.-K., Jung D. (2021). MNNG-Regulated Differentially Expressed Genes that Contribute to Cancer Development in Stomach Cells. Korean J. Clin. Lab. Sci..

[34] Kamineni A., Williams M.A., Schwartz S.M., Cook L.S., Weiss N.S. (1999). The Incidence of Gastric Carcinoma in Asian Migrants to the United States and Their Descendants. Cancer Causes Control.

[35] Taylor V.M., Ko L.K., Hwang J.H., Sin M.-K., Inadomi J.M. (2015). Gastric Cancer in Asian American Populations: A Neglected Health Disparity. Asian Pac. J. Cancer Prev..

[36] Red Meat, Processed Meat and Cancer. https://www.cancercouncil.com.au/1in3cancers/lifestyle-choices-and-cancer/red-meat-processed-meat-and-cancer/.

[37] Zhu H., Yang X., Zhang C., Zhu C., Tao G., Zhao L., Tang S., Shu Z., Cai J., Dai S. (2013). Red and Processed Meat Intake Is Associated with Higher Gastric Cancer Risk: A Meta-Analysis of Epidemiological Observational Studies. PLoS ONE.

[38] Borucka A., Ostaszewski K. (2008). Koncepcja Resilience. Kluczowe Pojęcia i Wybrane Zagadnienia. Med. Wieku. Rozw..

[39] Kim J.H., Lee J., Choi I.J., Kim Y.-I., Kwon O., Kim H., Kim J. (2018). Dietary Carotenoids Intake and the Risk of Gastric Cancer: A Case—Control Study in Korea. Nutrients.

[40] Chen Q.-H., Wu B.-K., Pan D., Sang L.-X., Chang B. (2021). Beta-Carotene and Its Protective Effect on Gastric Cancer. World J. Clin. Cases.

[41] Liu C., Russell R.M. (2008). Nutrition and Gastric Cancer Risk: An Update. Nutr. Rev..

[42] Tsugane S., Sasazuki S. (2007). Diet and the Risk of Gastric Cancer: Review of Epidemiological Evidence. Gastric. Cancer.

[43] Agudo A., Cayssials V., Bonet C., Tjønneland A., Overvad K., Boutron-Ruault M.-C., Affret A., Fagherazzi G., Katzke V., Schübel R. (2018). Inflammatory Potential of the Diet and Risk of Gastric Cancer in the European Prospective Investigation into Cancer and Nutrition (EPIC) Study. Am. J. Clin. Nutr..

[44] Wroblewski L.E., Peek R.M., Wilson K.T. (2010). *Helicobacter Pylori* and Gastric Cancer: Factors That Modulate Disease Risk. Clin. Microbiol. Rev..

[45] *Helicobacter Pylori* and Cancer—NCI. https://www.cancer.gov/about-cancer/causes-prevention/risk/infectious-agents/h-pylori-fact-sheet.

[46] Ishaq S., Nunn L. (2015). *Helicobacter Pylori* and Gastric Cancer: A State of the Art Review. Gastroenterol. Hepatol. Bed Bench.

[47] Díaz P., Valenzuela Valderrama M., Bravo J., Quest A.F.G. (2018). *Helicobacter pylori* and Gastric Cancer: Adaptive Cellular Mechanisms Involved in Disease Progression. Front. Microbiol..

[48] Choi I.J., Kim C.G., Lee J.Y., Kim Y.-I., Kook M.-C., Park B., Joo J. (2020). Family History of Gastric Cancer and *Helicobacter Pylori* Treatment. New Engl. J. Med..

[49] Yang J., Zhou X., Liu X., Ling Z., Ji F. (2021). Role of the Gastric Microbiome in Gastric Cancer: From Carcinogenesis to Treatment. Front. Microbiol..

[50] Yang P., Zhou Y., Chen B., Wan H.-W., Jia G.-Q., Bai H.-L., Wu X.-T. (2009). Overweight, Obesity and Gastric Cancer Risk: Results from a Meta-Analysis of Cohort Studies. Eur. J. Cancer.

[51] Hirabayashi M., Inoue M., Sawada N., Saito E., Abe S.K., Hidaka A., Iwasaki M., Yamaji T., Shimazu T., Shibuya K. (2019). Effect of Body-Mass Index on the Risk of Gastric Cancer: A Population-Based Cohort Study in A Japanese Population. Cancer Epidemiol..

[52] Perre A. Battling Obesity and Cancer? An Integrated Team? S Approach—Onco’Zine 2018. https://www.oncozine.com/battling-obesity-and-cancer-an-integrated-teams-approach/.

[53] Cui X., Zhang H., Cao A., Cao L., Hu X. (2020). Cytokine TNF-α Promotes Invasion and Metastasis of Gastric Cancer by down-Regulating Pentraxin3. J. Cancer.

[54] Kinoshita H., Hirata Y., Nakagawa H., Sakamoto K., Hayakawa Y., Takahashi R., Nakata W., Sakitani K., Serizawa T., Hikiba Y. (2013). Interleukin-6 Mediates Epithelial–Stromal Interactions and Promotes Gastric Tumorigenesis. PLoS ONE.

[55] Liu J.-F., Chen P.-C., Chang T.-M., Hou C.-H. (2020). Monocyte Chemoattractant Protein-1 Promotes Cancer Cell Migration via c-Raf/MAPK/AP-1 Pathway and MMP-9 Production in Osteosarcoma. J. Exp. Clin. Cancer Res..

[56] Menéndez P., Padilla D., Villarejo P., Menéndez J.M., Lora D. (2012). Does Bariatric Surgery Decrease Gastric Cancer Risk?. HepatoGastroenterol. Hepatol. Bed Bench..

[57] Aurello P., Petrucciani N., Antolino L., Giulitti D., D’Angelo F., Ramacciato G. (2017). Follow-up after Curative Resection for Gastric Cancer: Is it Time to Tailor it?. World J. Gastroenterol..

[58] Lahner E., Annibale B. (2009). Pernicious Anemia: New Insights from a Gastroenterol Point of View. World J. Gastroenterol..

[59] Cisło M., Filip A.A., Arnold Offerhaus G.J., Ciseł B., Rawicz-Pruszyński K., Skierucha M., Polkowski W.P. (2018). Distinct Molecular Subtypes of Gastric Cancer: From Laurén to Molecular Pathology. Oncotarget.

[60] Park H.S., Lee S.-Y., Hong S.N., Kim J.H., Sung I.-K., Park H.S., Shim C.S., Jin C.J. (2013). Early Gastric Cancer-Like Advanced Gastric Cancer Versus Advanced Gastric Cancer-Like Early Gastric Cancer. Clin. Endosc..

[61] De Sol A., Trastulli S., Grassi V., Corsi A., Barillaro I., Boccolini A., Di Patrizi M.S., DI Rocco G., Santoro A., Cirocchi R. (2014). Requirement for a Standardised Definition of Advanced Gastric Cancer. Oncol. Lett..

[62] Machlowska J., Baj J., Sitarz M., Maciejewski R., Sitarz R. (2020). Gastric Cancer: Epidemiology, Risk Factors, Classification, Genomic Characteristics and Treatment Strategies. Int. J. Mol. Sci..

[63] Carneiro F. Pathology of Gastric Cancer. 38. https://oncologypro.esmo.org/content/download/240507/4025970/file/2019-ESMO-Preceptorship-Gastric-Pathology-Fatima-Carneiro.pdf.

[64] Friis-Hansen L. (2006). Achlorhydria is Associated with Gastric Microbial Overgrowth and Development of Cancer: Lessons Learned from the Gastrin Knockout Mouse. Scand. J. Clin. Lab. Investig..

[65] Berlth F., Bollschweiler E., Drebber U., Hoelscher A.H., Moenig S. (2014). Pathohistological Classification Systems in Gastric Cancer: Diagnostic Relevance and Prognostic Value. World J. Gastroenterol..

[66] Al-Shamsi H.O., Alzaabi A.A., Afrit M., Abu-Gheida I., Musallam K.M. (2021). Clinicopathological Features of Gastric Cancer in a Cohort of Gulf Council Countries’ Patients: A Cross-Sectional Study of 96 Cases. J. Oncol Res. Rev. Rep..

[67] The Cancer Genome Atlas—Gastric Adenocarcinoma Study—NCI. https://www.cancer.gov/about-nci/organization/ccg/research/structural-genomics/tcga/studied-cancers/stomach.

[68] Sohn B.H., Hwang J.-E., Jang H.-J., Lee H.-S., Oh S.C., Shim J.-J., Lee K.-W., Kim E.H., Yim S.Y., Lee S.H. (2017). Clinical Significance of Four Molecular Subtypes of Gastric Cancer Identified by The Cancer Genome Atlas Project. Clin. Cancer Res..

[69] Serra O., Galán M., Ginesta M.M., Calvo M., Sala N., Salazar R. (2019). Comparison and Applicability of Molecular Classifications for Gastric Cancer. Cancer Treat. Rev..

[70] Batalha S., Ferreira S., Brito C. (2021). The Peripheral Immune Landscape of Breast Cancer: Clinical Findings and In Vitro Models for Biomarker Discovery. Cancers.

[71] Shibata D., Weiss L.M. (1992). Epstein-Barr Virus-Associated Gastric Adenocarcinoma. Am. J. Pathol..

[72] Alarcón A., Figueroa U., Espinoza B., Sandoval A., Carrasco-Aviño G., Aguayo F.R., Corvalan A.H. (2017). Epstein-Barr Virus–Associated Gastric Carcinoma: The Americas’ Perspective.

[73] Tang C.-T., Zeng L., Yang J., Zeng C., Chen Y. (2020). Analysis of the Incidence and Survival of Gastric Cancer Based on the Lauren Classification: A Large Population-Based Study Using SEER. Front Oncol..

[74] Macke L., Schulz C., Malfertheiner P. (2021). The Fear of Gastric Cancer in Patients with Dyspepsia: Challenge in Specialist Care Gastroenterology. Dig. Dis..

[75] Choi M. (2012). Gastric Cancer Screening and Alarm Symptoms in Early Gastric Cancer.

[76] Maconi G., Kurihara H., Panizzo V., Russo A., Cristaldi M., Marrelli D., Roviello F., de Manzoni G., Di Leo A., Morgagni P. (2003). Gastric Cancer in Young Patients with no Alarm Symptoms: Focus on Delay in Diagnosis, Stage of Neoplasm and Survival. Scand. J. Gastroenterol..

[77] Stephens M.R., Lewis W.G., White S., Blackshaw G.R.J.C., Edwards P., Barry J.D., Allison M.C. (2005). Prognostic Significance of Alarm Symptoms in Patients with Gastric Cancer. J. Br. Surg..

[78] Bowrey D.J., Griffin S.M., Wayman J., Karat D., Hayes N., Raimes S.A. (2006). Use of Alarm Symptoms to Select Dyspeptics for Endoscopy Causes Patients with Curable Esophagogastric Cancer to Be Overlooked. Surg. Endosc..

[79] Rasmussen S., Haastrup P.F., Balasubramaniam K., Christensen R.D., Søndergaard J., Jarbøl D.E. (2018). Predictive Values of Upper Gastrointestinal Cancer Alarm Symptoms in the General Population: A Nationwide Cohort Study. BMC Cancer.

[80] Dos Santos Guedes M.T., de Jesus J.P., de Souza Filho O., Fontenele R.M., Sousa A.I. (2014). Clinical and Epidemiological Profile of Cases of Deaths from Stomach Cancer in the National Cancer Institute, Brazil. Ecancermedicalscience.

[81] Mao L., Franke J. (2015). Symbiosis, Dysbiosis, and Rebiosis—The Value of Metaproteomics in Human Microbiome Monitoring. Proteomics.

[82] Sheflin A.M., Whitney A.K., Weir T.L. (2014). Cancer-Promoting Effects of Microbial Dysbiosis. Curr. Oncol. Rep..

[83] Nasr R., Shamseddine A., Mukherji D., Nassar F., Temraz S. (2020). The Crosstalk between Microbiome and Immune Response in Gastric Cancer. Int. J. Mol. Sci..

[84] Hao W.-L., Lee Y.-K., Spencer J.F.T., de Spencer A.L.R. (2004). Microflora of the Gastrointestinal Tract. Public Health Microbiol.ogy: Methods and Protocols.

[85] O’May G.A., Reynolds N., Macfarlane G.T. (2005). Effect of PH on an In Vitro Model of Gastric Microbiota in Enteral Nutrition Patients. Appl. Environ. Microbiol..

[86] Martinez J.E., Kahana D.D., Ghuman S., Wilson H.P., Wilson J., Kim S.C.J., Lagishetty V., Jacobs J.P., Sinha-Hikim A.P., Friedman T.C. (2021). Unhealthy Lifestyle and Gut Dysbiosis: A Better Understanding of the Effects of Poor Diet and Nicotine on the Intestinal Microbiome. Front. Endocrinol..

[87] Caguazango J.C. (2020). Ecological Models of Gastric Microbiota Dysbiosis: *Helicobacter Pylori* and Gastric Carcinogenesis. Med. Microecol..

[88] Ferreira R.M., Pereira-Marques J., Pinto-Ribeiro I., Costa J.L., Carneiro F., Machado J.C., Figueiredo C. (2018). Gastric Microbial Community Profiling Reveals a Dysbiotic Cancer-Associated Microbiota. Gut.

[89] Wang L., Zhou J., Xin Y., Geng C., Tian Z., Yu X., Dong Q. (2016). Bacterial Overgrowth and Diversification of Microbiota in Gastric Cancer. Eur. J. Gastroenterol. Hepatol..

[90] Griffin M., Shakespeare S., Shepherd H.J., Harding C.J., Létard J.-F., Desplanches C., Goeta A.E., Howard J.A.K., Powell A.K., Mereacre V. (2011). A Symmetry-Breaking Spin-State Transition in Iron(III). Angew. Chem. Int. Ed..

[91] Hatakeyama M. (2014). *Helicobacter Pylori* CagA and Gastric Cancer: A Paradigm for Hit-and-Run Carcinogenesis. Cell Host Microbe.

[92] Vinasco K., Mitchell H.M., Kaakoush N.O., Castaño-Rodríguez N. (2019). Microbial Carcinogenesis: Lactic Acid Bacteria in Gastric Cancer. Biochim. Biophys. Acta Rev. Cancer.

[93] Li Z.-P., Liu J.-X., Lu L.-L., Wang L.-L., Xu L., Guo Z.-H., Dong Q.-J. (2021). Overgrowth of Lactobacillus in Gastric Cancer. World J. Gastrointest. Oncol..

[94] Whiteside S.A., Mohiuddin M.M., Shlimon S., Chahal J., MacPherson C.W., Jass J., Tompkins T.A., Creuzenet C. (2021). In Vitro Framework to Assess the Anti-*Helicobacter Pylori* Potential of Lactic Acid Bacteria Secretions as Alternatives to Antibiotics. Int. J. Mol. Sci..

[95] Ping W., Senyan H., Li G., Yan C., Long L. (2018). Increased Lactate in Gastric Cancer Tumor-Infiltrating Lymphocytes is Related to Impaired T Cell Function Due to MiR-34a Deregulated Lactate Dehydrogenase, A. Cell. Physiol. Biochem..

[96] Aday U., Tatlı F., Akpulat F.V., İnan M., Kafadar M.T., Bilge H., Başol Ö., Oğuz A. (2020). Prognostic Significance of Pretreatment Serum Lactate Dehydrogenase-to-Albumin Ratio in Gastric Cancer. Contemp. Oncol..

[97] Chattopadhyay I., Gundamaraju R., Jha N.K., Gupta P.K., Dey A., Mandal C.C., Ford B.M. (2022). Interplay between Dysbiosis of Gut Microbiome, Lipid Metabolism, and Tumorigenesis: Can Gut Dysbiosis Stand as a Prognostic Marker in Cancer?. Dis. Markers.

[98] Pappas-Gogos G., Tepelenis K., Fousekis F., Katsanos K., Pitiakoudis M., Vlachos K. (2022). The Implication of Gastric Microbiome in the Treatment of Gastric Cancer. Cancers.

[99] Jaye K., Li C.G., Chang D., Bhuyan D.J. (2022). The Role of Key Gut Microbial Metabolites in the Development and Treatment of Cancer. Gut Microbes.

[100] Mertowska P., Mertowski S., Wojnicka J., Korona-Głowniak I., Grywalska E., Błażewicz A., Załuska W. (2021). A Link between Chronic Kidney Disease and Gut Microbiota in Immunological and Nutritional Aspects. Nutrients.

[101] Mirzaei R., Afaghi A., Babakhani S., Sohrabi M.R., Hosseini-Fard S.R., Babolhavaeji K., Khani Ali Akbari S., Yousefimashouf R., Karampoor S. (2021). Role of Microbiota-Derived Short-Chain Fatty Acids in Cancer Development and Prevention. Biomed. Pharmacother..

[102] Coussens L.M., Werb Z. (2002). Inflammation and Cancer. Nature.

[103] Singh N., Baby D., Rajguru J.P., Patil P.B., Thakkannavar S.S., Pujari V.B. (2019). Inflammation and Cancer. Ann. Afr. Med..

[104] Zhao H., Wu L., Yan G., Chen Y., Zhou M., Wu Y., Li Y. (2021). Inflammation and Tumor Progression: Signaling Pathways and Targeted Intervention. Sig. Transduct. Target Ther..

[105] Oya Y., Hayakawa Y., Koike K. (2020). Tumor Microenvironment in Gastric Cancers. Cancer Sci..

[106] Kim T.S., da Silva E., Coit D.G., Tang L.H. (2019). Intratumoral Immune Response to Gastric Cancer Varies by Molecular and Histologic Subtype. Am. J. Surg. Pathol..

[107] Colombo M.P., Piconese S. (2007). Regulatory T-Cell Inhibition versus Depletion: The Right Choice in Cancer Immunotherapy. Nat. Rev. Cancer.

[108] Zhang Y., Lazarus J., Steele N.G., Yan W., Lee H.-J., Nwosu Z.C., Halbrook C.J., Menjivar R.E., Kemp S.B., Sirihorachai V.R. (2020). Regulatory T-Cell Depletion Alters the Tumor Microenvironment and Accelerates Pancreatic Carcinogenesis. Cancer Discov..

[109] Wang Y.-Q., Zhang Y., Jiang W., Chen Y.-P., Xu S.-Y., Liu N., Zhao Y., Li L., Lei Y., Hong X.-H. (2019). Development and Validation of an Immune Checkpoint-Based Signature to Predict Prognosis in Nasopharyngeal Carcinoma Using Computational Pathology Analysis. J. Immunother. Cancer.

[110] Zhang X., Pan Z. (2020). Influence of Microbiota on Immunity and Immunotherapy for Gastric and Esophageal Cancers. Gastroenterol. Rep..

[111] Meng C., Bai C., Brown T.D., Hood L.E., Tian Q. (2018). Human Gut Microbiota and Gastrointestinal Cancer. Genom. Proteom. Bioinform..

[112] Cadamuro A.C.T., Rossi A.F.T., Maniezzo N.M., Silva A.E. (2014). *Helicobacter Pylori* Infection: Host Immune Response, Implications on Gene Expression and MicroRNAs. World J. Gastroenterol. WJG.

[113] Chang W.-L., Yeh Y.-C., Sheu B.-S. (2018). The Impacts of H. Pylori Virulence Factors on the Development of Gastroduodenal Diseases. J. Biomed. Sci..

[114] Wen S., Moss S.F. (2009). *Helicobacter Pylori* Virulence Factors in Gastric Carcinogenesis. Cancer Lett..

[115] Foegeding N.J., Caston R.R., McClain M.S., Ohi M.D., Cover T.L. (2016). An Overview of *Helicobacter Pylori* VacA Toxin Biology. Toxins.

[116] Caston R.R., Sierra J.C., Foegeding N.J., Truelock M.D., Campbell A.M., Frick-Cheng A.E., Bimczok D., Wilson K.T., McClain M.S., Cover T.L. (2020). Functional Properties of *Helicobacter Pylori* VacA Toxin M1 and M2 Variants. Infect. Immunity.

[117] Djekic A., Müller A. (2016). The Immunomodulator VacA Promotes Immune Tolerance and Persistent *Helicobacter Pylori* Infection through its Activities on T-Cells and Antigen-Presenting Cells. Toxins.

[118] Stein M., Ruggiero P., Rappuoli R., Bagnoli F. (2013). *Helicobacter Pylori* CagA: From Pathogenic Mechanisms to Its Use as an Anti-Cancer Vaccine. Front. Immunol..

[119] Takahashi-Kanemitsu A., Knight C.T., Hatakeyama M. (2020). Molecular Anatomy and Pathogenic Actions of *Helicobacter Pylori* CagA That Underpin Gastric Carcinogenesis. Cell Mol. Immunol..

[120] Li W., Zhou Y., Shang C., Sang H., Zhu H. (2020). Effects of Environmental PH on the Growth of Gastric Cancer Cells. Gastroenterol. Res. Pract..

[121] Hardbower D.M., Peek R.M., Wilson K.T. (2014). At the Bench: *Helicobacter Pylori*, Dysregulated Host Responses, DNA Damage, and Gastric Cancer. J. Leukoc. Biol..

[122] Sayed I.M., Sahan A.Z., Venkova T., Chakraborty A., Mukhopadhyay D., Bimczok D., Beswick E.J., Reyes V.E., Pinchuk I., Sahoo D. (2020). *Helicobacter Pylori* Infection Downregulates the DNA Glycosylase NEIL2, Resulting in Increased Genome Damage and Inflammation in Gastric Epithelial Cells. J. Biol. Chem..

[123] Mahdi B.M. (2014). Immune Response to Helicobacter Pylori.

[124] Könönen E., Gursoy U.K. (2022). Oral Prevotella Species and Their Connection to Events of Clinical Relevance in Gastrointestinal and Respiratory Tracts. Front. Microbiol..

[125] Zhang X., Li C., Cao W., Zhang Z. (2021). Alterations of Gastric Microbiota in Gastric Cancer and Precancerous Stages. Front. Cell. Infect. Microbiol..

[126] Barra W.F., Sarquis D.P., Khayat A.S., Khayat B.C.M., Demachki S., Anaissi A.K.M., Ishak G., Santos N.P.C., dos Santos S.E.B., Burbano R.R. (2021). Gastric Cancer Microbiome. Pathobiology.

[127] Gunathilake M.N., Lee J., Choi I.J., Kim Y.-I., Ahn Y., Park C., Kim J. (2019). Association between the Relative Abundance of Gastric Microbiota and the Risk of Gastric Cancer: A Case-Control Study. Sci. Rep..

[128] Montalban-Arques A., Wurm P., Trajanoski S., Schauer S., Kienesberger S., Halwachs B., Högenauer C., Langner C., Gorkiewicz G. (2016). Propionibacterium Acnes Overabundance and Natural Killer Group 2 Member D System Activation in Corpus-Dominant Lymphocytic Gastritis. J. Pathol..

[129] Chen Y., Chen B., Yang T., Xiao W., Qian L., Ding Y., Ji M., Ge X., Gong W. (2017). Human Fused NKG2D–IL-15 Protein Controls Xenografted Human Gastric Cancer through the Recruitment and Activation of NK Cells. Cell. Mol. Immunol..

[130] Liu X., Shao L., Liu X., Ji F., Mei Y., Cheng Y., Liu F., Yan C., Li L., Ling Z. (2019). Alterations of Gastric Mucosal Microbiota across Different Stomach Microhabitats in a Cohort of 276 Patients with Gastric Cancer. EBioMedicine.

[131] Signat B., Roques C., Poulet P., Duffaut D. (2011). Fusobacterium Nucleatum in Periodontal Health and Disease. Curr. Issues Mol. Biol..

[132] Boehm E.T., Thon C., Kupcinskas J., Steponaitiene R., Skieceviciene J., Canbay A., Malfertheiner P., Link A. (2020). Fusobacterium Nucleatum Is Associated with Worse Prognosis in Lauren’s Diffuse Type Gastric Cancer Patients. Sci. Rep..

[133] Hsieh Y.-Y., Tung S.-Y., Pan H.-Y., Chang T.-S., Wei K.-L., Chen W.-M., Deng Y.-F., Lu C.-K., Lai Y.-H., Wu C.-S. (2021). Fusobacterium Nucleatum Colonization is Associated with Decreased Survival of *Helicobacter Pylori*-Positive Gastric Cancer Patients. World J. Gastroenterol..

[134] Safdar A., Rolston K.V. (2007). Stenotrophomonas Maltophilia: Changing Spectrum of a Serious Bacterial Pathogen in Patients with Cancer. Clin. Infect. Dis..

[135] Ling Z., Shao L., Liu X., Cheng Y., Yan C., Mei Y., Ji F., Liu X. (2019). Regulatory T Cells and Plasmacytoid Dendritic Cells within the Tumor Microenvironment in Gastric Cancer Are Correlated with Gastric Microbiota Dysbiosis: A Preliminary Study. Front. Immunol..

[136] Guo Q., Qin H., Liu X., Zhang X., Chen Z., Qin T., Chang L., Zhang W. (2022). The Emerging Roles of Human Gut Microbiota in Gastrointestinal Cancer. Front. Immunol..

[137] Matson V., Chervin C.S., Gajewski T.F. (2021). Cancer and the Microbiome—Influence of the Commensal Microbiota on Cancer, Immune Responses, and Immunotherapy. Gastroenterology.

[138] AL-Ishaq R.K., Koklesova L., Kubatka P., Büsselberg D. (2022). Immunomodulation by Gut Microbiome on Gastrointestinal Cancers: Focusing on Colorectal Cancer. Cancers.

